# Microbial butyrate capacity is reduced in inflamed mucosa in patients with ulcerative colitis

**DOI:** 10.1038/s41598-024-54257-9

**Published:** 2024-02-12

**Authors:** Sushrut Jangi, John Moyer, Sarah Sandlow, May Fu, Hannah Chen, Ann Shum, Katie Hsia, Laura Cersosimo, Vladimir Yeliseyev, Naisi Zhao, Lynn Bry, Dominique S Michaud

**Affiliations:** 1 Department of Medicine, Tufts Medical Center, Boston, MA USA; 2 Pathology and Laboratory Medicine, Tufts Medical Center, Boston, MA USA; 3 Department of Pathology, Brigham and Women’s Hospital, 221 Longwood Ave, Boston, MA USA; 4 Public Health and Community Medicine, Tufts University School of Medicine, Boston, Ma USA; 5 Proger 3, Division of Gastroenterology, Tufts Medical Center, 800 Washington Street, Boston, MA 02111 USA

**Keywords:** Inflammatory bowel disease, Mucosal mycobiome, Short chain fatty acids, Gastroenterology, Microbial communities

## Abstract

Reduced butyrate-production capacity has been reported in fecal microbial communities in patients with active ulcerative colitis. However, the butyrate-production capacity of the mucosal microbiome from active vs quiescent mucosa in ulcerative colitis has been unexplored. We sought to determine the diversity and relative abundance of mucosal bacterial and fungal communities from endoscopically active vs quiescent mucosa in patients with UC, and aimed to predict contributions of mucosal microbial communities to butyrate synthesis. Systematic, segmental right- and left-sided biopsies were obtained from endoscopically active (n = 13) or quiescent (n = 17) colonic mucosa, among 15 patients with pan-colonic ulcerative colitis. Dietary fiber intake of patients was performed using the validated five-item FiberScreen questionnaire. Amplicon sequencing of mucosal bacteria and fungi was performed. The diversity and relative abundance of mucosal bacterial and fungal taxa were quantified, and predicted contributions to butyrate synthesis were ascertained. Bacterial alpha and beta diversity were similar between active vs quiescent mucosa. Butyrogenic taxa were significantly increased in quiescence, including *Butyricimonas*, *Subdoligranulum*, and *Alistipes*. Predicted butyrate kinase activity was significantly and concomitantly increased in quiescent mucosa. Fiber intake was positively correlated with butyrogenic microbes. Compared to mucosal bacterial prevalence, mucosal fungi were detected in low prevalence. Butyrogenic microbes are relatively increased in quiescent mucosa in ulcerative colitis, and may be related to increased fiber intake during quiescence. Manipulation of the mucosal microbiome towards butyrate-producing bacteria may be associated with endoscopic quiescence.

## Introduction

Ulcerative colitis (UC) is a form of inflammatory bowel disease (IBD) that primarily affects the colon, leading to frequent relapses and increased lifetime morbidity^1^. The pathogenesis of UC is driven by disruptions in host-microbiome homeostasis, with alterations in specific taxa and microbial products linked to UC inflammatory status^2–4^.

Gut microbiome studies in UC have primarily been performed utilizing fecal samples, a suboptimal proxy for characterizing the biogeographical variation of microbes throughout the gastrointestinal tract. Mucosa-associated microbiota can directly interact with host epithelium and reshape mucosal immune responses, therefore may have greater relevance for pathogenesis in UC^5,6^. Furthermore, the segmental distribution of the mucosal microbiota may differentially affect host immune microenvironments^7^. Mucosal *Clostridium, Bifidobacterium* and *Lactobacilli* can induce gastrointestinal regulatory T cells^8,9^, while mucosal segmental filamentous bacteria and even mucosal fungi can induce colonic Th17 cells^6,10^. The potential of mucosal microbes to regulate inflammatory responses in the colon has been associated with microbial production of short-chain fatty acids (SCFAs), primarily butyrate, a dietary fiber metabolite^11^. However, few studies have evaluated the biogeography of the mucosal bacteriome and mycobiome, mucosal SCFA production potential, and colonic inflammation in patients with UC.

We examined the mucosal microbiome across 30 segments of active or quiescent colonic mucosa among 15 patients with UC. We evaluated the microbial biogeography of both bacterial and fungal mucosal compartments utilizing high-throughput 16 s rDNA bacterial sequencing and 18 internal transcribed spacer (ITS2) fungal sequencing, and predicted mucosal microbial gene contributions to butyrate-production potential.

## Materials and methods

### Study cohort

We prospectively identified 15 patients with previously diagnosed pan-colonic ulcerative colitis who were planned for routine disease assessment at Tufts Medical Center, a tertiary referral hospital in downtown Boston, MA. Included patients had to have a history of endo-histologically documented pan-colitis, with no exposure to antibiotics or probiotics within 3 months of colonoscopy. All patients fasted for 24 h and received 4 L of Golytely colon cleansing prep prior to colonoscopy.

### Clinical metadata

Clinical assessment of inflammatory activity at time of colonoscopy was performed using the 12-point Mayo score. Endoscopic evaluation was performed during colonoscopy by a single gastroenterologist (SJ), an IBD-trained practitioner in Mayo endoscopic scoring. Eight biopsies were obtained in the right colon and left colon per patient, targeted to the worst affected area. For each patient, the most severe Mayo endoscopic score (MES) per segment was used to categorize an endoscopic severity category for the right and left colon. The Mayo endoscopic subscore (MES) was graded endoscopically as either 0 (normal colon), 1 (erythema, blurring of vascular pattern), 2 (friability, absence of vascular pattern, or erosions), or 3 (spontaneous bleeding or ulcers). Two pathologists (MF, HC) blinded to endoscopic results prospectively assigned a modified Nancy Histologic Index score (NHI)^12^ to the most severe histologic segment for the right and left colon. Fiber intake was quantified using the 2-week validated five-item FiberScreen questionnaire, that assessed fruit, vegetable, whole-grain, nut, and legume consumption^13^.

### Mucosal sample collection and storage

Mucosal samples were obtained using forceps biopsy as described in the prior section. All mucosal samples were handled using sterile forceps and were immediately snap frozen in cryovials in a cooler maintained at 0 °C. Samples were transported at this temperature to a biorepository at Tufts Medical Center, where they were immediately frozen at – 20 °C. Following collection of all sample accruals, samples were simultaneously thawed for DNA isolation.

### Bacterial isolation, fungal isolation, and library prep

DNA was extracted and purified with the Quick-DNA Fecal/Soil Microbe Miniprep kit (Zymo Research, Irvine, CA). Cell lysis was performed with a Vortex Genie-2 for 40 min per manufacturer’s suggestions. 16S rRNA and ITS sequencing libraries were generated with the Quick-16S Plus NGS Library Prep Kit and Quick-ITS Plus NGS Library Prep Kit, respectively (Zymo Research, Irvine, CA). The 16S rRNA V3-V4 hypervariable region was targeted with the primers 341F (mixture of CCTACGGGDGGCWGCAG, CCTAYGGGGYGCWGCAG) and 806R-GACTACNVGGGTMTCTAATCC) and the ITS rRNA with ITS3f. GCATCGATGAAGAACGCAG and ITS4r-TCCTCCGCTTATTGATATGC (Zymo Research, Irvine, CA). The ZymoBIOMICS Microbial DNA Standard, a mixture of bacterial and fungal DNA was used the positive amplification control. Candida albicans was used as a positive extraction and additional amplification control for ITS rRNA. The ZymoBIOMICS ITS qPCR community standard was used to generate a standard curve (7.5 × 106, 7.5 × 104, and 7.5 × 102 ITS copies/µl) for the detection of the ITS in the samples. Samples below the limit of detection (Ct ≥ 35) were negative for ITS and were not sequenced. The 16S rRNA and ITS amplicon libraries were pooled and subsequently sequenced using the MiSeq v3 kit with 600 cycles (Illumina, San Diego, CA). For ITS, a total of 21 samples were sequenced, 16 samples produced > 50 K raw reads per sample, 5 had under 5000 raw reads and were subsequently removed from the data set. Greater than 90 K raw reads were generated per 16S rRNA sample. To validate results of *Candida* mucosal fungal sequencing, quantitative PCR was performed using Norgen’s *Candida*-specific primers in triplicate on the Quant Studio 12 Flex.

### Outcomes

Outcomes included the alpha, beta diversity, relative abundance, and predicted butyrate synthesis of the mucosal bacteriome and mucosal mycobiome in endoscopically active vs quiescent mucosa.

### Analyses

#### Diversity and relative abundance

Alpha diversity of the microbiome was assessed using Shannon and Chao1 diversity indices. For beta-diversity, amplicon sequence variant count data was transformed using centered log ratio transformation, and the robust Aitchison distance was utilized, appropriate for compositional data^14^. To determine the relative abundance of microbes, we excluded rare organisms, defined as taxa found in < 5% of samples.

### Butyrate synthesis predictions

PICRUSt2^15^ was utilized to predict butyrate production potential based on taxonomic information. The abundance of the terminal enzyme in the butyrate synthesis pathway (butyrate kinase) was assessed, which is typically used as a biomarker for butyrate-producing communities^16,17^. Additionally, using PICRUST2, we assessed predicted activity in the acetyl CoA to butanoate pathway and the lysine to butanoate pathway.

### Correlations between fiber intake and mucosal microbes

Correlation analysis was performed to assess for correlations between fiber intake and the relative abundance of significantly altered mucosal taxa. Fiber intake was quantified by assigning a Fiber Score based on the validated fiber survey tool^18^.

### Statistics

To model alpha diversity, a linear mixed effects model was utilized and adjusted for right- or left- sided segments obtained from the same patient. A Wilcoxon rank-sum test was used to evaluate whether alpha diversity indices differed significantly between comparators. To evaluate significance for beta-diversity, permutational ANOVA analysis was used to calculate significance between groups (p < 0.05). For differential abundance analysis, adjustment for repeated measures was performed when colonic mucosal samples were from different segments within the same patient, by modeling subject as a random effect^19^. P-values were corrected for multiple testing using the Benjamini and Hochberg method (reported as q-values)^20^.

### Ethics approval

Ethical approval for the study was obtained from the Tufts Medical Center Institutional Review Board, Boston, MA. The study was performed in accordance with the ethical standards as laid down in the 1964 Declaration of Helsinki and its later amendments or comparable ethical standards.

### Consent

Informed consent was obtained from all participants.

## Results

### Study cohort

Fifteen patients with UC underwent colonoscopy for routine disease assessment. Demographic characteristics are provided in Table 1. All patients had a history of endo-histologically documented pan-colitis. No patients were exposed to antibiotics or probiotics within 3 months of sample collection. A high fiber dietary pattern (FiberScreen Score $\ge$ 5) was followed by 57% of the cohort, while a low fiber dietary pattern (FiberScreen Score < 5) was followed by 43% of the cohort. At the time of endoscopic assessment, 5/15 had endoscopic inflammation throughout the colon, 1/15 had only right-sided endoscopic inflammation, 2/15 had only left-sided endoscopic inflammation, and 7/15 were in complete endoscopic quiescence.

**Table 1:** Clinical cohort.

		Total Cohort
n		15
Age, mean (SD)		40 (14)
Gender, female, frequency		6 (40%)
Disease extent, n, (%)	E1	0
	E2	0
	E3	15 (100%)
Disease duration, years, median (IQR)		14 (7.5–18.5)
Rectal bleeding score, median (IQR)		0 (0–1.5)
Stool frequency score, mean (SD)		2 (1–2)
Endoscopic activity		8 (53.3%)
Endo-histologic activity		8 (53.3%)
Fiber intake score		5 (4–7.25)
Immunosuppressive exposure, frequency (%)		11 (73.3%)
Concurrent steroid use		4 (26.7%)
Concurrent antibiotic use		0
Concurrent probiotic use		0

Biopsies were obtained from the right and left colon in each patient. Among 30 colonic segments obtained, 17 segments demonstrated endoscopic quiescence (MES = 0 or 1) and 13 segments demonstrated moderate-severe endoscopic inflammation (MES $\ge$ 2) (Table 2). Among endoscopically quiescent segments, 3/17 demonstrated mild neutrophilic infiltration, with 14/17 demonstrating non-active histology. Among endoscopically active segments, 13/13 samples demonstrated mild-severe histologic neutrophilic infiltration. Concordance of endoscopic and histologic assessment was 90% (Table 2).

**Table 2:** Characteristics of active vs quiescent mucosa.

	Active segments	Quiescent segments
n	13	17
Right-sided	6	9
Left-sided	7	8
Mayo endoscopic score, median, IQR	2 (2–2)	0 (0–1)
Histologic activity, frequency (%)	13 (100%)	3 (17.6%)
FiberScore, median (IQR)	4 (3.8–5)	5 (4.8–8.3)

### Mucosal bacterial diversity and relative abundance

**Figure 1:**
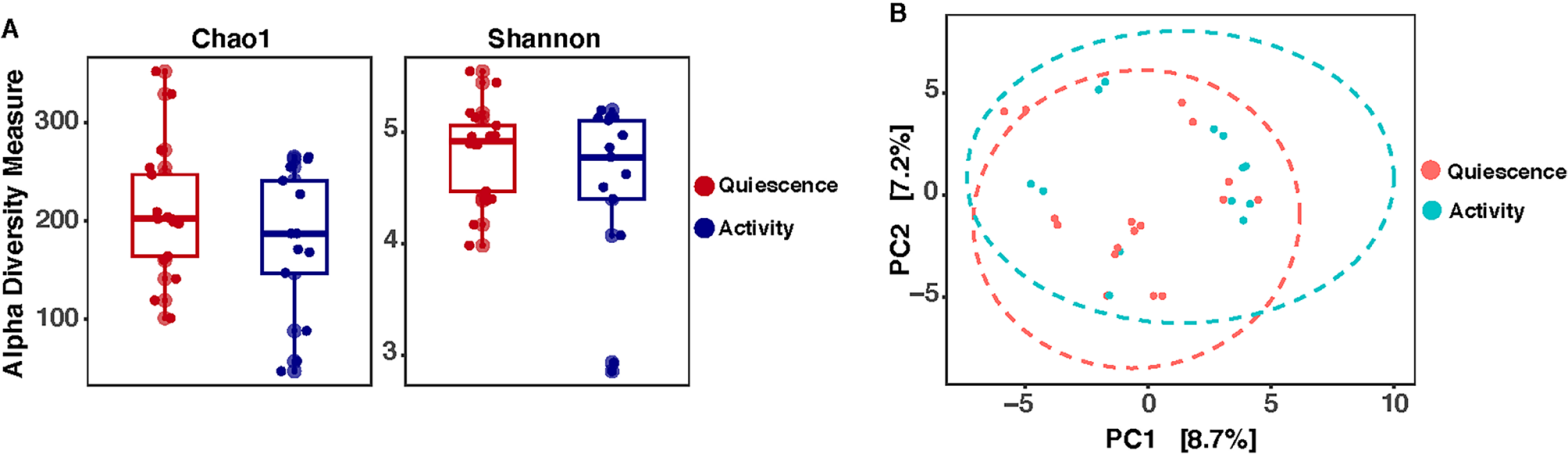
Bacterial diversity in colonic mucosa among patients with active (blue) vs quiescent (red) ulcerative colitis. (**A**) Alpha diversity with Chao1 richness and Shannon diversity index (y-axis) (**B**) Beta diversity using centered log ratio transformation and the robust Aitchison distance.

Bacterial alpha diversity did not differ between endoscopically quiescent (n = 13) and endoscopically active (n = 17) mucosa (Shannon diversity index, 4.8 vs 4.5, p = 0.74). Bacterial beta diversity was also similar between endoscopically quiescent vs active mucosa (p = 0.13, PERMANOVA) (Fig. 1A,B).

**Figure 2:**
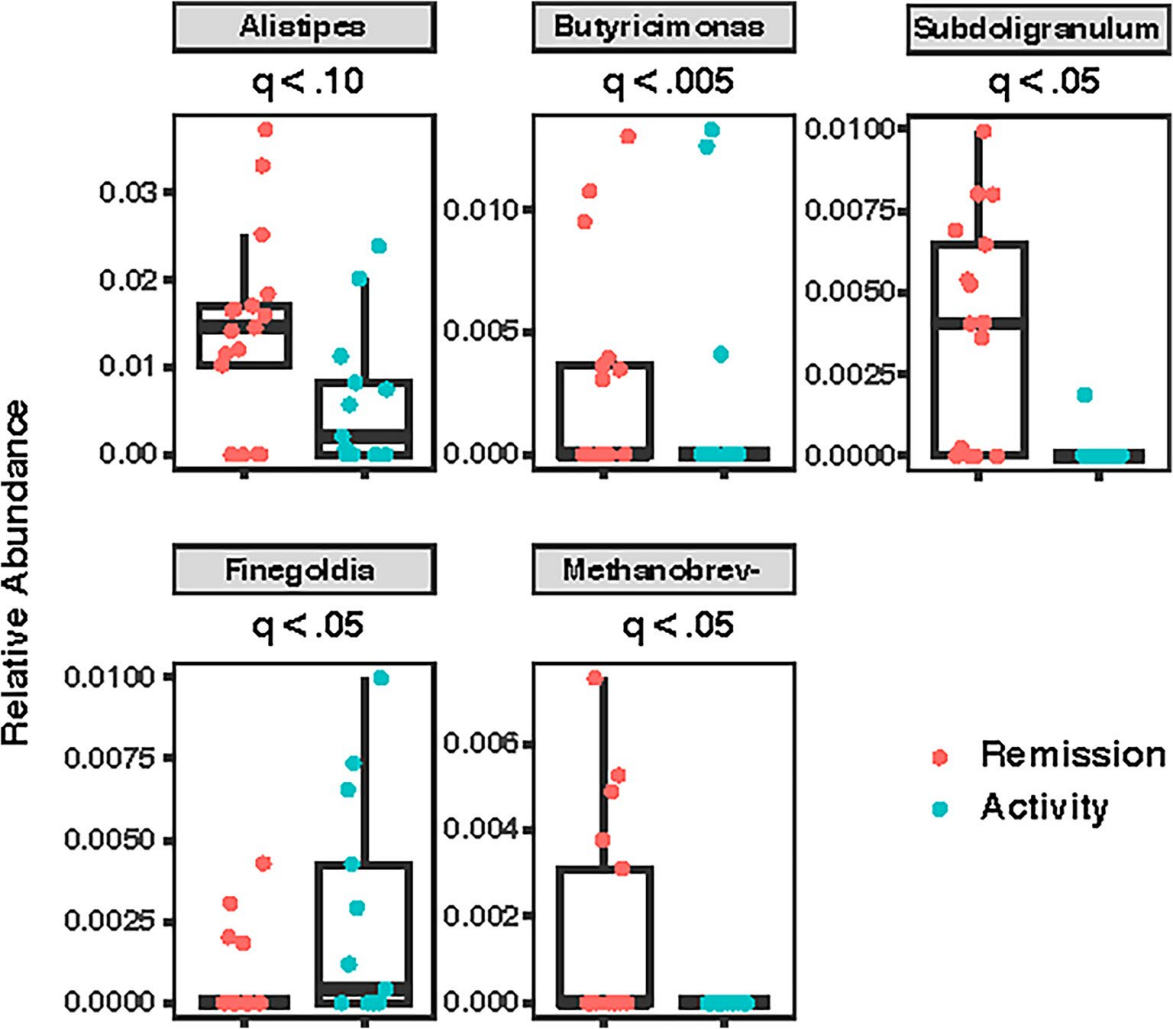
Bacterial relative abundance in colonic mucosa among patients with active (green) vs quiescent (red) colitis. Differential abundance was calculated using a negative binomial model (DESeq2) with Benjamini–Hochberg method to control for multiple comparisons.

Quiescent mucosa had a relative increase in several butyrate-producing bacterial genera including *Butyricimonas* (q-val < 0.005), *Subdoligranulum* (q-val < 0.05), and *Alistipes* (q-val = 0.1). In endoscopically active mucosa, *Finegoldia* (q-val < 0.05) was relatively increased [Fig. 2].

Within the right colon exclusively, during endoscopic quiescence (n = 9/15), there were relative increases in mucosal *Ruminococcus* (q-val < 0.05), *Catenibacterium* (q-val < 0.05), and *Methanobrevibacter* (q-val < 0.05), compared to during inflammation (n = 6/15) (Supp Fig. 1a). In the left colon, during inflammation (n = 7/15), there were relative increases in family *Enterobacteriaceae* (q-val < 0.005), genus *Eubacterium* (q-val < 0.005), *Bacillus* (q-val < 0.05), and *Corynebacterium (*q-val < 0.05). During quiescence in the left colon (n = 8/15), *Subdoligranulum* was relatively increased (q-val < 0.005), compared to during activity (Supp Fig. 1b).

### Predicted mucosal butyrate synthesis activity

**Figure 3:**
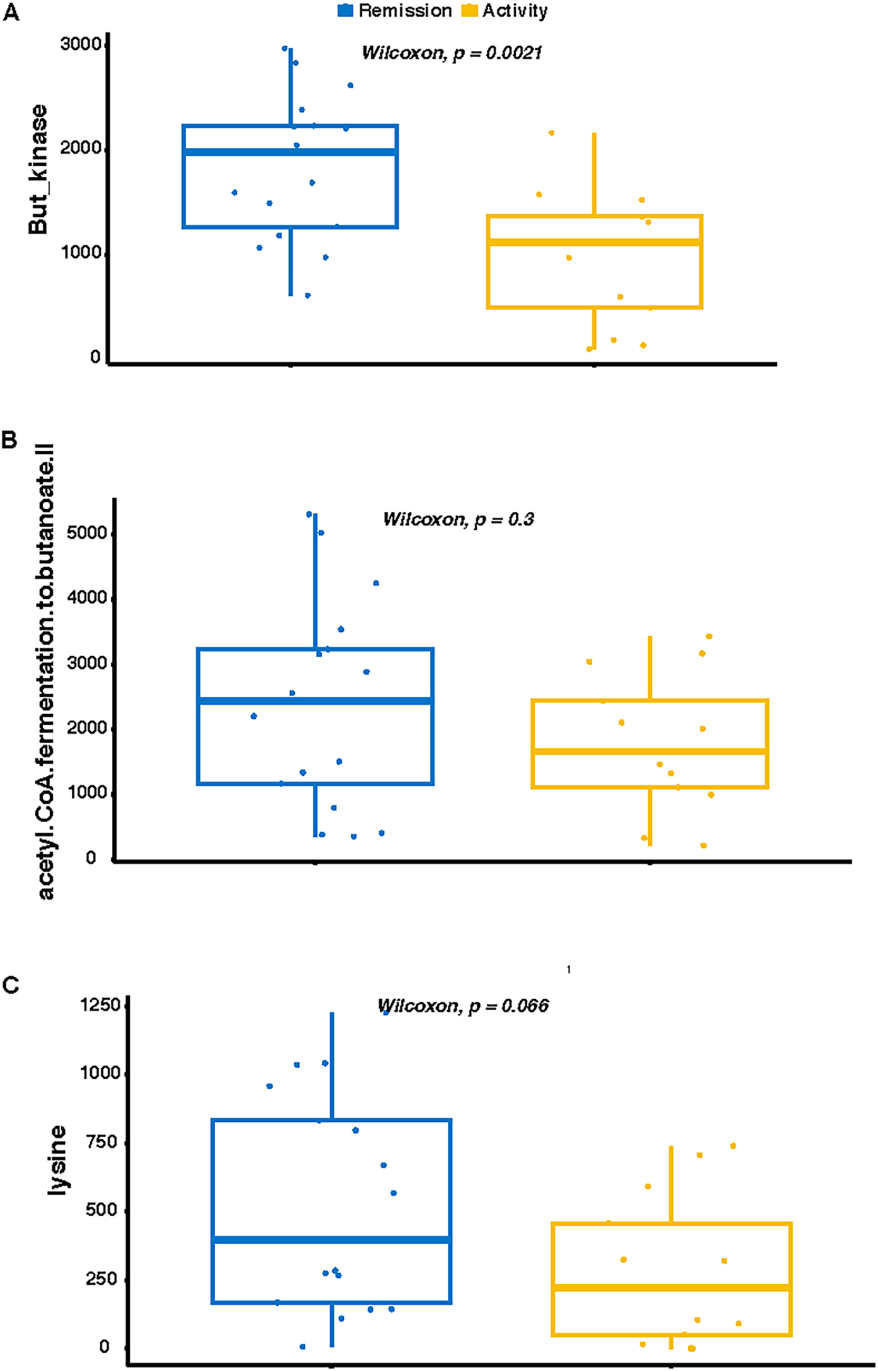
Predicted butyrate synthesis by colonic microbes. Picrust2 was used to infer butyrate metabolic pathway activity between patients with active (yellow) vs quiescent (blue) ulcerative colitis as assessed by relative abundance of (**A**) butyrate kinase enzyme (**B**) acetyl CoA to butyrate pathway (**C**) lysine to butyrate pathway.

Butyrate kinase activity was significantly more abundant in microbes from endoscopically quiescent vs endoscopically active mucosa (1847.8 vs 975.1, p < 0.005) (Fig. 3a). The acetyl CoA to butanoate pathway was also numerically increased in microbes from endoscopically quiescent mucosa vs active mucosa, although that difference was not significant (2387.4 vs 1793.4, p = 0.30) (Fig. 3b). Similarly, the lysine to butanoate was numerically increased in microbes from endoscopically quiescent vs active mucosa (525.6 vs 279.5, p = 0.06), although this did not reach statistical significance thresholds (Fig. 3c).

Given that dietary fiber can favor colonic butyrate production, correlations between fiber intake and the relative abundance of mucosal taxa associated with quiescence vs inflammation were assessed. Elevated fiber intake was positively correlated with relative abundance of butyrate-producers *Butyricimonas*, *Subdoligranulum*, *Alistipes*, *Ruminococcus*, and *Catenibacterium*—genera associated with endoscopic quiescence (Supp Fig. 2). Reduced fiber intake was correlated with the relative abundance of genera associated with inflammation, including *Finegoldia*, *Enterobacteriaceae*, and *Corynebacterium* (Supp Fig. 2).

### Mucosal fungal microbiome during endoscopic activity and quiescence

Diversity of the fungal mucosal microbiome was lower than bacterial diversity. Fungal alpha diversity was similar in endoscopically active and quiescent mucosa (Shannon diversity index, 0.68 vs 0.50, p = 0.70). Beta diversity was also similar between the two environments (PERMANOVA, p = 0.95) (Supp Fig. 3A,B).

Fungal genera were found sparingly in the mucosa of patients with active or quiescent UC, including genera from phylum *Ascomycota* (*Meyerozyma*, *Saccharomyces*, *Aureobasidium*, *Aspergillus*, and *Pencillium* and family *Didymellaceae*) and from phylum *Basidiomycota* (*Malassezia, Coprinellus*, *Perenniporia*, and *Typhula*). No sequences attributed to *Candida albicans* were found in the gut mucosa. Due to the prior reported association between fecal *Candida* and inflammatory activity in UC^4,21,22^, quantitative PCR was utilized to try to detect *Candida* using *Candida*-specific primers, but this analysis confirmed that no mucosal *Candida* could be detected in any samples despite positive *Candida* controls (Supp Table 1). The distribution and relative abundance of each mucosal fungal taxa across patients is presented in (Table 3). Given the low prevalence of fungal taxa, differential abundance testing was not performed.

**Table 3:** Distribution of fungal microbes in colonic mucosa in patients with ulcerative colitis.

Phylum	Genus	Distri- bution	Inflammatory status	Prevalence (%)	Relative abundance (%)
Ascomycota	Meyerozyma	Right colon	Quiescence	18	3
	Saccharomyces	Pan-colonic	Active and quiescent	18	5
	Aureobasidium	Pan-colonic	Active and quiescent	27	7
	Aspergillus	Pan-colonic	Active and quiescent	36	19
	Penicillium	Right colon	Active	9	3.8
	f_Didymellaceae	Pan-colonic	Active and quiescent	36	17.4
Basidiomycota	Malassezia	Pan-colonic	Active and quiescent	27	6.4
	Coprinellus	Pan-colonic	quiescent	18	4.6
	Perenniporia	Pan-colonic	Active and quiescent	18	15.7
	Typhula	Pan-colonic	Active and quiescent	45	10.7
	Unassigned	–	–	63	7.5

## Discussion

Among 30 mucosal segments across 15 patients with UC, quiescent mucosa was associated with an increase in several butyrate-producing taxa, including *Butyricimonas*, *Subdoligranulum*, and *Alistipes*. Coincident with these findings, higher predicted levels of microbial butyrate kinase were detected from quiescent mucosal samples relative to active mucosa.

Our findings that butyrate-production potential is increased in quiescent mucosa in UC extends prior reports that stool from patients in remission also have increased butyrate-production potential^23–25^, although comparisons between active vs quiescent UC—especially in the mucosal compartment—have been limited^26^. Hirano et al. obtained paired mucosal biopsies from 14 patients with left-sided colitis and compared microbial communities in inflamed (rectum) vs non-inflamed (transverse) colon. They found relative increases in genus *Cloacibacterium* and family *Tissierellaceae*, with reductions in genus *Neisseria* in the inflamed rectum, which were not reported in our study. Overall, the microbes they identified are low abundance organisms, and it remains possible that differences in microgeography (they utilized patients with left-sided disease vs pancolitis) or methodology (they may have utilized a lower total mucosal yield than in our experiments) may explain this difference. Others have found increases in *Bacteroides* or *Faecalibacterium* in quiescent mucosa, both of which can demonstrate high butyrate-production capacity^26,27^. One study utilizing mucosal brushings have also identified reductions in butyrogenic bacterial groups in endoscopically active tissue^28^. While variability in the underlying microbial groups may differ across studies, the functional niche of these microbes are similar, suggesting that a functional metagenomic studies of mucosal metacommunities is needed.

Dietary data in this study supports that fiber intake correlated with increases in these same groups of butyrate-producers, suggesting that fiber may mediate the associations between observed microbes and disease status. In experimental mouse models of colitis, mice fed high-fiber diets demonstrated microbiota-dependent increases in luminal butyrate concentrations, with less severe colitis^29^. In patients with UC, a single study reported that high-fiber intake led to increased fecal butyrate-producing bacteria, with improved clinical outcomes^30^. However, the influence of fiber intake on the mucosal microbiome and inflammatory activity are unknown. Larger studies examining the relationship between fiber intake and the mucosal microbiome among patients with UC in complete remission, for example, can further answer the question of whether fiber intake may be associated with an altered mucosal microbial community with increases in butyrate capacity and improved clinical outcomes.

Furthermore, we provide evidence that fungal taxa are also detectable on the mucosal surfaces of patients with UC, albeit with smaller read counts compared to bacteria. In contrast to a single prior report^31^, we found no evidence of *Candida*–previously linked in the stool to inflammatory activity in UC^4^—in any mucosal samples, confirmed both by ITS2 sequencing and qPCR utilizing specific primers directed against *Candida*. Studies of the mucosal mycobiome are sparse in patients with UC. In a single study of 10 patients with active UC, investigators found that *Candida* composed 6% of total reads. However, it remains unclear how many of these patients actually had *Candida* detectable. Similar to our cohort, they also found *Pencillium* and *Malassezia* in the mucosal mycobiome^32^. Assessment of mucosal fungal species may require bulk surgical specimens to improve yield for adequate detection. Understanding the mucosal mycobiome is warranted in UC, especially as specific fungi, such as *Candida* are known to form biofilms with bacteria^33^, which may play a pathogenic role in UC^34^. Future studies employing either surgical specimens or repeated and targeted biopsies of endoscopically visible biofilms, with use of scanning electron microscopy and confocal microscopy may provide further insight into fungal-bacterial mucosal interactions in patients with UC.

Bacterial communities may also affect the dynamics of fungal populations in the mucosa. For example, butyrate-producing bacteria are known to antagonize *Candida* and limit its growth^35^. Unfortunately, metagenomic studies of the mycobiome are currently limited by the relatively small number of fungal genes in metagenomic reference catalogues, with only about 0.1% of 3.3 million reference genes of eukaryotic origin^36^. While Picrust2 and other software packages such as FunFun^37^ can provide some insight into functional capacity of fungal sequences, until more comprehensive reference genes are included, mucosal mycobial metagenomic studies will be difficult to perform.

Our study was limited by the small sample size and its cross-sectional nature. We also did not have the statistical power to adjust for the effect of medications on our study results. However, our patients were prospectively recruited, included patients with pan-ulcerative colitis, utilized validated indices of clinical, endoscopic, and histological activity, and included a validated survey tool for assessment of short-term fiber intake. Additionally, we focused on the mucosal bacteriome and mycobiome, which is less well-represented in microbiome studies, and assessed variability by segment in the right and left colon. Overall, our report supports that butyrate-producing mucosal bacteria are increased in quiescent mucosa relative to active mucosa, with predicted increases in microbial butyrate kinase activity, and associations with increased fiber intake. As exogenous butyrate has had mixed results in treating patients with UC, efforts to modulate the mucosal microbiome to increase butyrate-production capacity may prove more useful for inducing remission. This study provides further evidence that butyrogenesis may be enhanced in quiescent mucosa. Microbial butyrate production in mucosally-adherent communities should continue to be evaluated as a potential treatment pathway towards remission outcomes in UC.

### Supplementary Information

Supplementary Information.

## Data Availability

The datasets generated and/or analysed during the current study are available in the Sequence Read Archive (SRA) repository. The accession number for the bacterial and fungal amplicon sequencing data is available using the Bioproject Accession number PRJNA1049435.

## Abbreviations

SCFAs: Short-chain fatty acids
ITS2: Internal transcribed spacer-2
MES: Mayo endoscopic score
