# Supplementary material for: Microbial butyrate capacity is reduced in inflamed mucosa in patients with ulcerative colitis
Source: Sci Rep. 2024 Feb 12;14:3479. doi: 10.1038/s41598-024-54257-9 (PMC10861456; doi:10.1038/s41598-024-54257-9)
Supplement: Supplementary file 1 — Supplementary Information. [file 41598_2024_54257_MOESM1_ESM.docx]

**Supp Fig 1 –** Correlations between FiberScore and relative abundance of significantly altered mucosal bacteria

**Supp Fig 2 -** Bacterial relative abundance in segmental colonic mucosa among patients with active (green) vs quiescent (red) colitis. Differential abundance was calculated using a negative binomial model (DESeq2) with Benjamini-Hochberg method to control for multiple comparisons in the (A) right colon (B) left colon.

**Supp Fig 3 -** Fungal diversity in colonic mucosa among patients with active (blue) vs quiescent (red) ulcerative colitis. (A) Alpha diversity with Chao1 richness and Shannon diversity index (y-axis) (b) Beta diversity using centered log ratio transformation and the robust Aitchison distance.

**Supp Fig 1**

**Supp Fig 2**

**Supp Fig 3**

**
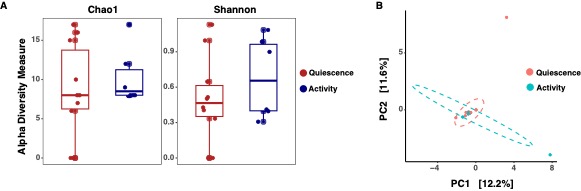
**

**Supp Table 1 – Quantitative polymerase chain reaction against mucosal *Candida albicans***

| **Sample** | **C. albicans Target (FAM)** | **PCR Control Target (VIC)** |
| --- | --- | --- |
| RTColon1 | not detected | detected |
| LTColon1 | not detected | detected |
| RTColon2 | not detected | detected |
| LTColon2 | not detected | detected |
| RTColon3 | not detected | detected |
| LTColon3 | not detected | detected |
| RTColon4 | not detected | detected |
| LTColon4 | not detected | detected |
| RTColon5 | not detected | detected |
| LTColon5 | not detected | detected |
| RTColon6 | not detected | detected |
| LTColon6 | not detected | detected |
| RTColon7 | not detected | detected |
| LTColon7 | not detected | detected |
| RTColon8 | not detected | detected |
| LTColon8 | not detected | detected |
| RTColon9 | not detected | detected |
| LTColon9 | not detected | detected |
| RTColon10 | not detected | detected |
| LTColon10 | not detected | detected |
| RTColon11 | not detected | detected |
| LTColon11 | not detected | detected |
| RTColon12 | not detected | detected |
| LTColon12 | not detected | detected |
| RTColon13 | not detected | detected |
| LTColon13 | not detected | detected |
| RTColon14 | not detected | detected |
| LTColon14 | not detected | detected |
| RTColon15 | not detected | detected |
| LTColon15 | not detected | detected |
| Candida albicans POS | detected | detected |
